# A novel development of a new single switch inductor coupled DC-DC converter for PV system with two-leg inverter

**DOI:** 10.1038/s41598-024-61637-8

**Published:** 2024-05-10

**Authors:** Shaik Moulana Samiulla, Kunamneni Rachananjali

**Affiliations:** Vignan’s Foundation for Science, Technology & Research, Guntur, Andhra Pradesh 522213 India

**Keywords:** Boost DC-DC converter, Optimal duty value, Fast MPP tracing speed, Good voltage gain, Less development cost, Plus easy handling, Engineering, Electrical and electronic engineering

## Abstract

From the power generation history**,** the nonrenewable power sources utilization is falling extremely because of their demerits are high atmospheric pollution, more expensive, more catchment area for development, high fossil fuel transportation cost, less flexibility, and reliability. So, the sunlight systems are utilized in this work for feeding the power to the central grid. On the earth, the sunlight energy availability is more and it is more flexible for the installation. However, the sunlight photovoltaic (PV) module’s power production is very low. To improve the power generation of the PV network, a modified slider maximum power point tracking (MPPT) controller is proposed in the first objective and it is interfaced with the sunlight system for capturing more sunlight insolation thereby moving the functioning point of the solar system from local MPP place to required global MPP place. The features of this sliding controller are continuous peak power production, easy development, less power dissipation losses, plus good dynamic system response. In the second objective, the available voltage of the PV is low which improved from low level to high level by utilizing the Wide voltage supply-inductor coupled converter. The development of this circuit needed very less inductive, plus capacitive components. Also, it is developed by selecting a single switch. As a result, the entire network power production cost is reduced. In the third objective, a two-leg inverter is proposed for the transformation of the DC voltage supply into three-phase powers. The MATLAB/Simulink tool is used to investigate the overall system.

## Introduction

At present, most of the power-selling companies working on renewable power source extraction to meet the automotive load demand. The present renewable energy networks are geothermal, solar, ocean energy, tidal networks, bioenergy, plus hydropower stations. In Ref.^1^, the geothermal power effectiveness is explained for the water irrigation application. Most of the geothermal power networks are installed in the US and Hawaii. In this power network, natural geothermal is availed in the presence of hot rocks, permeability underground, and fluids. Here, the fluid flows towards the geothermal network to produce the steam and this steam is sent to the generator-coupled turbine to produce electricity without any fluctuations. The geothermal network's features can function at 300 to 700°F, emits zero hazardous gasses, high internal complexity in building, a large amount of groundwater necessity, plus more functioning cost. The ocean power network works depends on the ocean water waves and its functioning temperature differences^2^. Here, high warmup water is pumped to the evaporator tank which consists of thermal fluids. The vaporized fluid runs the turbine to supply the energy to the high peak load conditions. The merits of ocean power networks are high energy potential, less emissions, good reliability, plus less impact on the natural conditions. But this system design cost is high, plus discontinuity in the energy production. The application of the ocean power system is the ability to work at emergency power requirement conditions^3^.

The wind power networks are utilized in the smart grid power production network for balancing the peak power demand. Here, the naturally available wind kinetic energy runs the turbine propeller which is coupled with the generator network. In this wind network, the air flows over the blades for achieving the lift which is similar to the effect on airplane wings. Most wind networks give more jobs to human beings, and it is a complete domestic energy source. Also, it is highly effective for the local communities, and cost-effective power production network when associated with the thermal power distribution network. The advantages of this system are zero CO_2_ emissions, more safety, a very simple structure, plus very easy operation. However, this network creates an impact on wildlife, with little noise, plus applicable for limited regions^4^. The bioenergy power generation happens by utilizing the biomass. In this network, the biomass is split into a small number of pieces and it is pumped to the boiler for burning the overall biomass. The biomass is converted into H_2_ thereby generating the utility power for automotive networks. The features of biomass networks are cleaner, fewer environmental pollutants, more silica, low heating value, higher moisture content, plus lower density^5^.

The major issue of the biomass network is nitrogen oxide which pollutes the ground level ozone layer. Also, it releases carbon monoxide directly into the atmosphere. So, hydro stations are coming into the market to limit the drawbacks of biomass systems. In this hydro station, the high head water storage is captured for running the hydro stations. The features of this system are the ability to control the frequent come floods, and it supplies energy at day as well as night time. The major use of this system is highly suitable for peak load demands^6^. However, the demerits of this system are expensive upfront costs, lack of available reservoirs, plus facility reliance on local hydrology. These all problems are mitigated by selecting the recently existing sunlight system. The sunlight systems are developed from the P-type and N-type material's electron flow. In the P, and N-type semiconductor materials, some free electrons absorb the sunlight photo energy for moving the low-level energy band to the high-level energy band^7^. The P material and N material combination form a PV cell. The PV cells are developed by utilizing the various categories of sunlight networks which are 1-diode, dual-diode, plus triple-diode dependent PV circuits. Here, in this work, the triple diode mathematical PV cell is developed by estimating its parameters by selecting the grasshopper optimization methodology^8^.

A single PV cell circuit produces only 0.97 V which is a very low-level voltage. So, there are several PV cells are organized in series, and parallel fashion to raise the power supply ability to the industrial and rural areas leaving human beings. This cell development has been done by selecting the following materials amor-Phou’s silicon, polysilicon, glass, plus silicon wafers. The silicon wafer is the most important primarily utilized material for implementing the polycrystalline sunlight system. In Ref.^9^, the manufacturers referred to the monocrystalline wafer for producing power at quick variations of atmospheric irradiation conditions. The manufacturing cost of the monocrystalline wafer is more when associated with the polycrystalline wafer. Also, the per unit power production of price the sunlight network is higher. So, the monocrystalline-based triple diode circuit sunlight system is developed in this work for extracting the more accurate I-V chrematistics. The features of this triple diode circuit system are more efficient, less distortions in the sunlight production power, a good fill factor, plus more flexibility for any environmental conditions^10^. However, the nonlinearity concept reduces the sunlight system efficiency. There are various models of power electronic converter circuits involved in the PV output circuit for balancing supply energy and consumer-utilized energy^11^.

From the recent power electronic networks, the converter circuits are differentiated based on the transformer involved and transformerless converter circuits. The isolated power circuits have the necessity of additional power rectifier circuits which are running with more installation regions. The flyback isolated topology is selected in Ref.^12^ for high-voltage-grade industrial applications. However, the flyback circuits have certain limitations which are more voltage stress, high ripples in the converter circuit voltage, low power density, plus low efficiency. Also, it faces the issue of more peak currents across the diodes and more complexity in controlling the entire converter circuit. The feedforward quadratic transformerless wide voltage rating converter circuit is developed in Ref.^13^ for battery charging applications in hydrogen-based electric vehicles. Also, it is also used in adapters, plus power distribution networks. The demerits of this circuit are a greater number of passive comments are used for developing the overall circuit, plus the driver circuit of this converter needed more ICs. As a result, the feedforward quadratic converter-based renewable power production network installation cost is very high. In the article^14^, there are more than two conventional converter circuits merged into one for supplying uniform energy to the automotive networks. In this interleaved circuit, there are three phases involved which work simultaneously whenever the emergency conditions occur.

**Figure 1:**
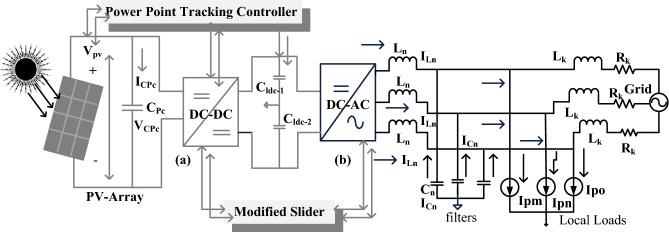
Overall, the steps involved power conversion system, (**a**) DC source, plus (**b**) AC source.

The Z-source wide power supply converter circuit is introduced in the work^15^ for solar/battery/wind smart grid power networks for balancing the voltage between the battery and wind power network. This converter performs the buck operation when the source energy is very high, and it enhances the power when the power consumption is greater. The features of the Z-source network are a greater range of DC load voltages, reduced in-rush ripple currents, plus good reliability. Especially, for renewable energy networks, this type of converter circuit gives wide voltage gain. But these type of converter networks produces discrete outputs, plus more starting current^16^. So, in this article, a single semiconductor device DC-DC converter circuit is introduced for balancing the load impedance with the sunlight system impedance thereby enhancing the power production capability of the sunlight network. Here, the couple inductor concept is proposed for the converter circuit for transferring the electrical power from one side of the common core to another side of the core. The common features of this type of converter are increased utilization of power electronic switches at low duty value conditions, and the voltage conversion ratio of the converter is enhanced by the continuous adjustment of transformer windings. So, the inductor-coupled network optimizes the functioning duty cycle of the converter as shown in Fig. 1.

**Figure 2:**
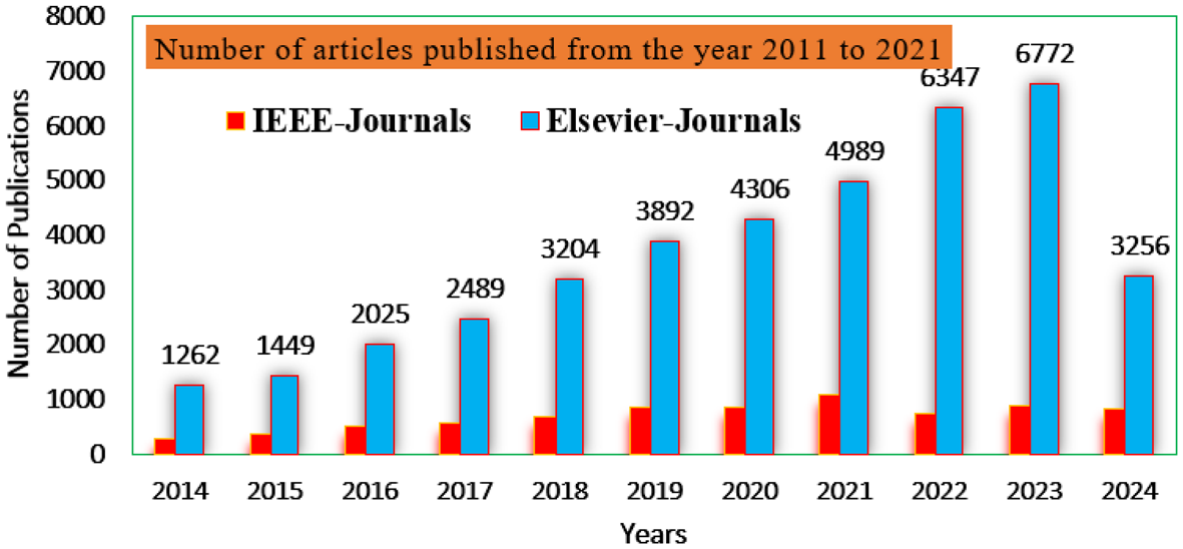
Power point tacking controllers’ publication status.

The sunlight network power completely works on the various atmospheric circumstances. To maintain the peak power availability from the sunlight system, the Perturb & Observe methodology is utilized in the wind/solar system for maintaining the grid voltage stability at multiple environmental conditions^17^. The present status of the MPPT methodologies is discussed in Fig. 2. Here, the considered step value on the sunlight network P–V curve is variable and it is increased step by step to reach the optimal duty value of the coupled inductor circuit DC-DC converter. The features of this controller are fast MPP catching speed, low convergence time, plus quick controller dynamic response. However, this controller supplies the fluctuated converter current with more heating losses. Also, it may not provide the exact MPP place. So, the conductance of the sunlight network is varied by integrating the incremental conductance method. This controller takes a slightly higher implementation cost when associated with the P&O concept. The limitations of both usual methods are compensated by choosing the lookup table power point identifier^18^. This method takes very low voltage rating components, plus easy handling at quick variations of sunlight conditions. However, these methods are not suitable for the shaded conditions of the sunlight network.

The artificial intelligence concept is illustrated in the article^19^ for balancing the voltage of the battery-interfaced wind energy network to supply uniform power to the automotive industry. The artificial controller’s development has been done by utilizing the working principle human brain. Here, each neuron is identified as one node and all the neurons are interfaced with the help of dendrites. The neurons collect the information from previously existing neurons for identifying the optimal solution for the nonlinear performance-based sunlight system. In the article^20^, the authors focused on the P&O with artificial intelligence MPPT controller for capturing the exact place of the microgrid-based wind/solar power production network. This hybrid algorithm development cost is low and reduces distortions across the functioning point of the sunlight network. Also, the flexibility, plus reliability of the overall wind/solar system are more by using this power point tracking controller. However, these controllers suffer from more power losses of the sunlight system under shading behavior conditions.

So, the adaptive modified slider MPPT methodology is developed in this article for running the overall system at peak power point conditions. The advantages of this proposed method when associated with the conventional controllers are easy operation, less complexity in understanding, low passive components necessary for developing the controller, and more suitability for the uniform and continuous changes of sunlight irradiation conditions. The available voltage of the inductors linked DC-DC converter is sent to the two-leg bridge converter for transferring the DC source into the AC source for supplying the energy to the local consumers, plus the utility grid. The remaining part of the work is organized as in Sect. “Modeling of proposed PV system and its description”, the mathematical development of the sunlight system cell, and its overall system description is mentioned. From Sect. “Proposed universal source voltage converter for PV”, the proposed converter voltage conversion ratio is obtained by adjusting the coupled inductor turns, and its power point identification by using modified MPPT methodology is mentioned in detail in Sect. “Small signal analysis of proposed slider controller”. The development of the introduced two-leg bridge inverter is discussed in Sect. “Development of power inverter circuit for solar PV”. Finally, the proposed sunlight network and its functioning strategy are explained in Sect. “Discussion of simulation results”. The conclusion of the proposed controller is given in Sect. “Conclusion”.

## Modeling of proposed PV system and its description

The sunlight network’s operating efficiency depends on its accurate nonlinear characteristics^21^. The one diode network development has been done based on the five constraints which are parallel resistance (R_Pt_), openly circuited PV network voltage (V_OcV_), ideality factor (β_n_), serially placed resistance (R_Sy_), plus PV output short-circuited current (I_Sci-n_). The merits of one diode network are a very simple structure, less iteration number required for identifying the optimal parameters of the sunlight system, less development cost, plus simple understanding. However, it may not generate pure I-V Characteristics of the sunlight network because its junction recombination operation effect is neglected. So, a dual diode concept is applied in the PV-integrated electric vehicle charging station to enhance the power production ability of the PV system. This model needed a few more variables because one more power semiconductor device is involved in the one diode circuit-based sunlight system which is diode reverse back current (I_Sci-m_), plus its related ideality parameter (β_m_). Here, all the junction’s reverse leakage currents are removed by involving one more diode in the dual diode-based sunlight network.

**Figure 3:**
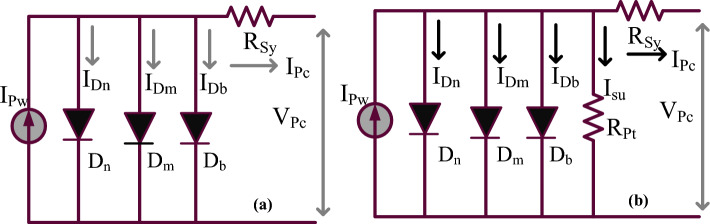
Utilized 3-diode PV, (**a**) Absence of shunt resistive component, plus (**b**) Including of R_Pt_.

**Figure 4:**
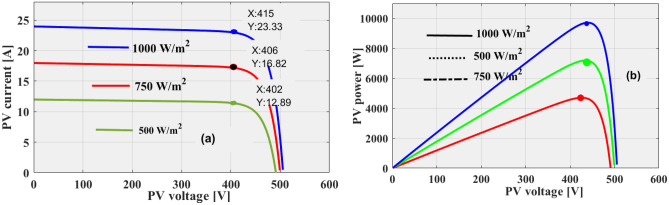
(**a**) Available I-V characteristics of PV system. (**b**) Available P–V characteristics of PV system.

Here, the proposed 3-diode configuration circuit needs overall nine constraints which are parallel resistance (R_Pt_), openly circuited PV network voltage (V_OcV_), ideality factors (β_n_, β_m_, plus β_b_), serially placed resistance (R_Sy_), plus PV output reverse short-circuited currents (I_Sci-n_, I_Sci-m_, plus I_Sci-b_). All the variables are properly identified by utilizing the various nature-inspired algorithms. From the literature study, the parameter’s identified algorithms are Jaya, Differential Evolutionary, wind drive optimization, plus soft computing methodologies. In this work, the 3-diode sunlight network parameters are obtained by applying the modified cuckoo search concept which is illustrated in Table 1. From Fig. 3), plus Fig. 3b, in the 1st circuit, the shunt resistive element is not taken into account because the reverse leakage current value is much less. However, in practical circuit working conditions, the shunt resistance comes in the sunlight system. So, the sunlight system generated current is represented as I_Pw_ and it is evaluated by selecting Eq. (1). The obtained V–I & P–V curves of the sunlight network are illustrated in Fig. 4a, and b.

**Table 1:** Considered variables for the implementation of triple diode-dependent PV network.

Parameters	Values
Selected solar peak power at quick variation of insolation’s (V_Pc_)	415.22 V
Peak current of sunlight network at variation of insolation’s (I_Pc_)	23.225 amps
Utilized power for the PV system at multiple temperatures (P_Pc_)	9.643 kWatts
Open PV circuit potential at variation of insolation’s (V_OCV_)	510.89 V
Short PV circuit potential at the variation of insolation’s (I_SCV_)	23.425 amps
Constant sunlight network output voltage thermal value	− 0.0486%/deg.c
Constant sunlight network output current thermal value for PV	0.05228%/deg.c
Working ideality factors of various power semiconductor diodes (β_n_, β_m_, plus β_b_)	0.8, 0.82, 0.81
Determined series placed sunlight system resistance value (R_Sy_)	0.3311 Ώ
Determined parallel placed sunlight system resistance value (R_Pt_)	374.998 Ώ
Photon current flow of sunlight network at multiple irradiations values (I_Pc_)	24.390 A
Currents of utilized diodes under saturation conditions (I_Sci-b_, I_Sci-m_, I_Sci-b_)	1.44 × 10^–10^ amps
Utilized sunlight insolation values for testing the proposed converter network (G)	1 k, 0.75 K, plus 0.5 KW/m^2^

1 $${{\text{I}}}_{{\text{Pc}}}={{\text{I}}}_{{\text{Pw}}}-{{\text{I}}}_{{{\text{D}}}_{{\text{n}}}}-{{\text{I}}}_{{{\text{D}}}_{{\text{m}}}}-{{\text{I}}}_{{{\text{D}}}_{{\text{b}}}},$$

2 $${{\text{I}}}_{{\text{Pc}}}={{\text{I}}}_{{\text{Pw}}}-{{\text{I}}}_{{{\text{Sci}}}_{{\text{n}}}}\left({{\text{e}}}^{\frac{{\text{q}}*\left({{\text{V}}}_{{\text{Pc}}}+{{\text{I}}}_{{\text{Pc}}}*{{\text{R}}}_{{\text{Sy}}}\right)}{{\upbeta }_{1}*{\text{KT}}}}-1\right)-{{\text{I}}}_{{{\text{Sci}}}_{{\text{m}}}}\left({{\text{e}}}^{\frac{{\text{q}}*\left({{\text{V}}}_{{\text{Pc}}}+{{\text{I}}}_{{\text{Pc}}}*{{\text{R}}}_{{\text{Sy}}}\right)}{{\upbeta }_{2}*{\text{KT}}}}-1\right)- {{\text{I}}}_{{{\text{Sci}}}_{{\text{b}}}}\left({{\text{e}}}^{\frac{{\text{q}}*\left({{\text{V}}}_{{\text{Pc}}}+{{\text{I}}}_{{\text{Pc}}}*{{\text{R}}}_{{\text{Sy}}}\right)}{{\upbeta }_{3}*{\text{KT}}}}-1\right),$$

3 $${{\text{I}}}_{{\text{Pw}}}={{\text{I}}}_{{\text{Pc}}}-{{\text{I}}}_{{\text{Dn}}}-{{\text{I}}}_{{\text{Dm}}}-{{\text{I}}}_{{\text{Db}}}-{{\text{I}}}_{{\text{pt}}},$$

4 $${{\text{I}}}_{{\text{Pc}}}={{\text{I}}}_{{\text{Pw}}}-{{\text{I}}}_{{{\text{Sci}}}_{{\text{n}}}}\left({{\text{e}}}^{\frac{{\text{q}}*\left({{\text{V}}}_{{\text{Pc}}}+{{\text{I}}}_{{\text{Pc}}}*{{\text{R}}}_{{\text{Sy}}}\right)}{{\upbeta }_{1}*{\text{KT}}}}-1\right)-{{\text{I}}}_{{{\text{Sci}}}_{{\text{m}}}}\left({{\text{e}}}^{\frac{{\text{q}}*\left({{\text{V}}}_{{\text{Pc}}}+{{\text{I}}}_{{\text{Pc}}}*{{\text{R}}}_{{\text{Sy}}}\right)}{{\upbeta }_{2}*{\text{KT}}}}-1\right)-{{\text{I}}}_{{\text{ptl}}},$$

5 $${{\text{I}}}_{{\text{Ptl}}}={{\text{I}}}_{{\text{Sci}}\_{\text{b}}}\left({{\text{e}}}^{\frac{{\text{q}}*({{\text{V}}}_{{\text{Pc}}}+{{\text{I}}}_{{\text{Pc}}}*{{\text{R}}}_{{\text{Sy}}})}{{\upbeta }_{3}*{\text{KT}}}}-1\right)+\frac{{{\text{V}}}_{{\text{Pc}}}+{{\text{I}}}_{{\text{Pc}}}{{\text{R}}}_{{\text{Sy}}}}{{{\text{R}}}_{{\text{pt}}}},$$

6 $${{\text{I}}}_{{\text{Sci}}\_{\text{n}}}={{\text{I}}}_{{\text{Sci}}\_{\text{m}}}={{\text{I}}}_{{\text{Sci}}\_{\text{b}}}={{\text{I}}}_{{\text{onc}}}* (\frac{{\text{T}}}{{{\text{T}}}_{{\text{onc}}}}{)}^{3}{\mathrm{ e}}^{\frac{{\text{q}}*{\text{Eg}}}{{\text{n}}*{\text{k}}}\left(\frac{1}{{{\text{T}}}_{{\text{onc}}}}-\frac{1}{{\text{T}}}\right)},$$

7 $${{\text{I}}}_{{\text{on}}}={{\text{I}}}_{{\text{on}}\_{\text{n}}}={{\text{I}}}_{{\text{on}}\_{\text{m}}}={{\text{I}}}_{{\text{on}}\_{\text{b}}}=\frac{{{\text{I}}}_{{\text{Sci}}\_{\text{n}}}}{{{\text{e}}}^{\left(\frac{{{\text{V}}}_{{\text{oc}}\_{\text{n}}}}{\upbeta *{{\text{V}}}_{*{\text{Tn}}}}\right)}}.$$

## Proposed universal source voltage converter for PV

**Figure 5:**
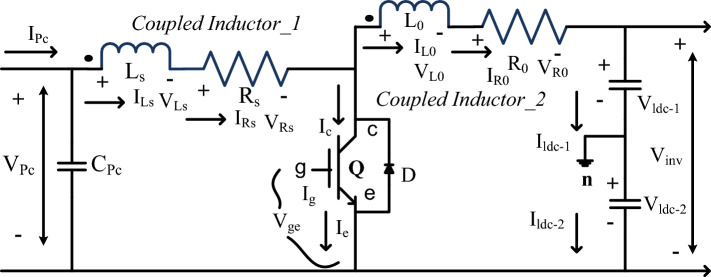
Proposed wide voltage conversion ratio power converter circuit.

The PV power network energy production required more higher installation cost. To limit this issue, there are various power converter network topologies are interlinked with the solar system for the production of peak voltages for the local load consumers^22^. As we know the isolated circuit topologies needed more passive components, and its manufacturing cost is also more. Here, the two inductors interlinked concept is utilized for moderate, plus more power automotive applications. This converter voltage conversion ratio is enhanced by the continuous adjustment of the transformer's two windings turns which are represented as N_Pr_, plus N_Se_. The turns ratio of the proposed converter is N_ti_ which is decided based on the secondary windings of transformer turns with associated the source winding turns. The working network of the proposed converter is illustrated in Fig. 5. From Fig. 5, the Insulated Gate Bipolar Transistor (IGBT) power semiconductor switch (Q) is selected for the development of the power converter circuit. The features of this switch are high source voltage control ability, high source impedance, low driver circuit cost, easy-to-make parallel operation, plus the ability to function above 200 °C temperature. Also, this switch's starting functioning speed is very high when associated with the other controllers.

Here, the sunlight source is directly sent to the converter network to improve the fill factor of the overall system. The dotted circle near the source inductor (L_s_), plus the resistor (R_s_) is defined as the current in flow. The currents, plus their related voltages flowing to the parameters L_s_, plus L_0_ are I_Ls_, I_L0_, V_Ls_, plus V_L0_. Similarly, for the variables (L_s_, plus L_0_) currents and voltages are I_Rs_, I_R0_, V_Rs_, plus V_R0_. The coupled capacitors parameters are identified as C_ldc-1_, plus C_ldc-2_, and their associated voltages and currents flowing through these elements are I_ldc-1_, I_ldc-2_, V_ldc-1_, plus V_ldc-2_. These DC-linked source voltages are interlined with the inverter input. The voltage conversion of the converter network is illustrated in Eq. (8). From Eq. (8), plus Eq. (10), the terminologies N_tura_, plus R_total_ are the overall inductors turns ratio and its related total resistance. The design constraints of the introduced converter circuit are discussed in Table 2.

**Table 2:** Design constraints of introduced DC-DC converter circuit for sunlight system.

S. no	Names of variables	Values
1.	Selected internally available inductive resistor (R_s_)	28.192 mΩ
2.	Inverter supply side capacitive element value (C_ldc-1_)	14.452 mF
3.	Inverter supply side capacitive element value (C_ldc-2_)	12.892 mF
4.	Inverter supply side inductive element value (L_0_)	22.7451 mH
5.	Sunlight system side capacitive element value (C_Pc_)	10.00 mF
6.	Sunlight system side inductive element value (L_s_)	14.55 mH
7.	Inverter supply side resistive element value (R_0_)	68.49 mΩ
8.	Overall resistive value of the converter circuit network (R_total_)	0.532 Ω
9.	Inverter output network filter circuit inductive value (L_n_)	10.99 mH
10.	Inverter output network filter circuit capacitive value (C_n_)	0.5921 mF
11.	Load side circuit transformer equivalent circuit inductive (L_k_)	0.88 mF
12.	Load side circuit transformer equivalent circuit resistor (R_k_)	0.878 mΩ

8 $$\frac{{{\text{V}}}_{{\text{inveter}}}}{{{\text{V}}}_{{\text{Pc}}}}=\frac{1+{{\text{N}}}_{{\text{tura}}}*\mathrm{Duty cycle}}{\mathrm{Duty cycle}},$$

9 $${{\text{L}}}_{{\text{Source}}}=\frac{{{\text{L}}}_{{\text{Overall}}}}{(1+{\mathrm{turns }({\text{N}})}^{2})},$$

10 $${{\text{L}}}_{{\text{out}}}=\frac{{\mathrm{turns }({\text{N}})}^{2}}{1+{\mathrm{turns }({\text{N}})}^{2}}*{{\text{L}}}_{{\text{total}}}={\mathrm{turns }\left({\text{N}}\right)}^{2}{{\text{L}}}_{{\text{S}}},$$

11 $${{\text{R}}}_{{\text{Source}}}=\frac{{{\text{R}}}_{{\text{toal}}}}{1+\mathrm{turns }({\text{N}})},$$

12 $${{\text{R}}}_{{\text{out}}}=\left(\frac{\mathrm{turns }({\text{N}})}{1+\mathrm{turns }({\text{N}})}\right)* {{\text{R}}}_{{\text{total}}.}$$

### Small signal analysis of proposed slider controller

**Figure 6:**
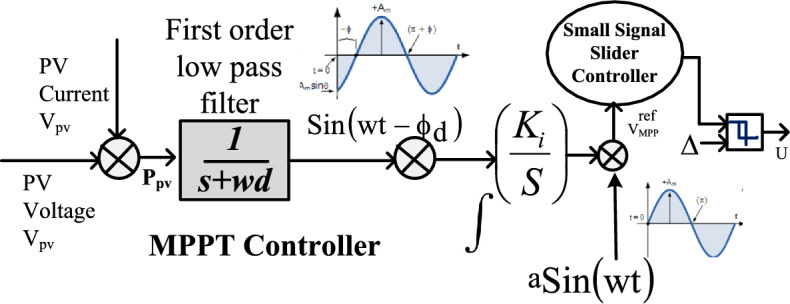
Solar power point tracker by using a modified slider controller.

In most of the existing power converter networks, there are more than two sensors are required. For all these sensing variables, the evaluation of state constraints is mandatory for identifying the duty pulses to the introduced coupled inductor converter network. In this proposed controller network, the error constants are enough for the effective operation of the converter network. From Fig. 6, the low-pass filter circuited is applied for transferring the direct current values to sinusoidal signals. Here, the low-pass network optimizes the entire sunlight system size. As a result, the cost, plus the required components for the development of the controller circuit is low. Also, this controller handles the nonlinear nature of the sunlight system very effectively thereby the converter circuit supplies wide supply voltage gain with low-level duty values. This controller tries to maintain uniform DC-link voltages at static irradiation as well as rapid variation of sunlight temperatures. In this section, the step-by-step process of the converter network development is discussed. Here, the straightforward inductor flux measurement is a quite tough task. So, the state constraints of inductors (I_x_) are used to determine the flux linkages of the inductors.

13 $${{\text{I}}}_{{\text{x}}}=\left\{\begin{array}{cc}{{\text{I}}}_{{\text{S}}}& {\text{T}}\in {{\text{T}}}_{\mathrm{switch \,on}}\\ {{\text{I}}}_{{\text{S}}}*\left(1+\mathrm{turns }({\text{N}})\right)& {\text{T}}\in {{\text{T}}}_{\mathrm{switch \,off}}\end{array}\right..$$

14 $$\frac{\Delta {{\text{I}}}_{{\text{x}}}}{\mathrm{\Delta t}}=\frac{{{\text{V}}}_{{\text{Pc}}}-{{\text{I}}}_{{\text{x}}}{\mathrm{ R}}_{{\text{x}}}}{{{\text{L}}}_{{\text{S}}}}+\left(\frac{{{\text{V}}}_{{\text{Pc}}}-{{\text{V}}}_{{\text{inv}}}}{{{\text{L}}}_{{\text{S}}}*\left(1+\mathrm{turns }({\text{N}})\right)}\left(1-\mathrm{\S }\right)\right)-{{\text{I}}}_{{\text{g}}},$$

15 $${{\text{I}}}_{{\text{g}}}=\left[\left(\frac{{{\text{R}}}_{{\text{S}}}+{{\text{R}}}_{{\text{o}}}}{{{\text{L}}}_{{\text{S}}}*{\left(1+\mathrm{turns }({\text{N}})\right)}^{2}}{{\text{I}}}_{{\text{x}}}\right)*\left(1-\mathrm{\S }\right)\right],$$

16 $$\frac{\Delta {{\text{V}}}_{{\text{inv}}}}{\mathrm{\Delta t}}=\left[\left(\frac{{{\text{I}}}_{{\text{x}}}}{\left(1+\mathrm{turns }({\text{N}})\right) {{\text{C}}}_{{\text{Pc}}}}\right)*\left(1-\mathrm{\S }\right)\right]-\frac{{{\text{I}}}_{{\text{inv}}}}{{{\text{C}}}_{{\text{Pc}}}}.$$

Here, the condition of the first state variable §$\in \left\{0, 1\right\}$ then the switching condition of the converter is mentioned in Eq. (17). The error variable state vector is illustrated in Eq. (18). The overall mathematical term of the converter is given in Eq. (19).

17 $$\begin{array}{*{20}c} {\S \, = { 1},{\text{ switching ON}}} \\ \,\,\,\,\,\,\,\,\,\,\,{ = \, 0,{\text{ switching OFF}}} \\ \end{array} ,$$

18 $${\text{Y}}=\left[{{\text{Y}}}_{1},{{\text{Y}}}_{2}\right]=\left[{{\text{I}}}_{{\text{x}}}-{{\text{I}}}_{{\text{x}}}^{{\text{refer}}},{{\text{V}}}_{{\text{inv}}}-{{\text{V}}}_{{\text{inv}}}^{{\text{refer}}}\right],$$

19 $$\dot{Y}={\text{Ey}}+{\text{Rz}}+{\text{Th}}+{\text{J}},$$

20 $${\text{E}}=\left[\begin{array}{cc}-\frac{({{\text{R}}}_{{\text{S}}}+{{\text{R}}}_{0})}{{{\text{L}}}_{{\text{S}}}{ \left(1+\mathrm{turn }({\text{N}})\right)}^{2}}& \frac{-1}{{{\text{L}}}_{{\text{S}}}*\left(1+\mathrm{turn }({\text{N}})\right)}\\ \frac{1}{{{\text{C}}}_{{\text{Pc}}}*\left(1+\mathrm{turn }({\text{N}})\right)}& 0\end{array}\right],\mathrm{ J}=\left[\begin{array}{c}\frac{{{\text{V}}}_{{\text{S}}}}{{{\text{L}}}_{{\text{S}}}*\left(1+\mathrm{turn }({\text{N}})\right)}\\ \frac{-{{\text{I}}}_{{\text{inv}}}}{{{\text{C}}}_{{\text{Pc}}}}\end{array}\right],$$

21 $${\text{R}}=\frac{{{\text{V}}}_{{\text{Pc}}}}{{{\text{L}}}_{S}}-\frac{{{\text{R}}}_{{\text{S}}}*{{\text{I}}}_{{\text{x}}}}{{{\text{L}}}_{{\text{S}}}}-\left(\frac{{{\text{V}}}_{{\text{S}}}-{{\text{V}}}_{{\text{o}}}}{{{\text{L}}}_{{\text{S}}}*\left(1+\mathrm{turn }\left({\text{N}}\right)\right)}\right)+\frac{{{\text{R}}}_{{\text{i}}}+{{\text{R}}}_{{\text{o}}}}{{{\text{L}}}_{{\text{i}}}*{\left(1+{\text{N}}\right)}^{2}}*{{\text{I}}}_{{\text{x}}}+\frac{{{\text{i}}}_{{\text{a}}}}{{\text{C}}*\left(1+{\text{N}}\right)},$$

22 $${\text{D}}=\left(\frac{1-\frac{{{\text{V}}}_{Pc}}{{{\text{V}}}_{{\text{Inv}}}}}{1+\frac{\mathrm{turn }({\text{N}})*{{\text{V}}}_{{\text{Pc}}}}{{{\text{V}}}_{{\text{inv}}}}}\right),$$

23 $${\text{S}}\left(f\right)={\mathrm{\pounds }}_{1}{{\text{y}}}_{1}+{\mathrm{\pounds }}_{2}{{\text{y}}}_{2}={\mathrm{\pounds }}^{{\text{T}}}{\text{y}}; {\mathrm{\pounds }}^{{\text{T}}}=\left[\begin{array}{cc}{\mathrm{\pounds }}_{1}& {\mathrm{\pounds }}_{2}\end{array}\right].$$

24 $$\left\{\begin{array}{cc}{S\left(\dot{f}\right)}<0& \begin{array}{cc}then& S\left(f\right)>0\end{array}\\ {S\left(\dot{f}\right)}>0& \begin{array}{cc}then& S\left(f\right)<0\end{array}\end{array}\right..$$

25 $$\mathrm{\S }=\left\{\begin{array}{ccc}\begin{array}{c}0\\ 1\end{array}& \begin{array}{c}then\\ then\end{array}& \begin{array}{c}S\left({\text{f}}\right)>0\\ S\left({\text{f}}\right)<0\end{array}\end{array}\right..$$

26 $$\left\{\begin{array}{c}{\uplambda }_{1}\left({\text{y}}\right)={\mathrm{\pounds }}^{{\text{T}}}Ey+{\mathrm{\pounds }}^{{\text{T}}}J<0,if S\left({\text{y}}\right)>0\\ {\uplambda }_{2}\left({\text{Y}}\right)={\mathrm{\pounds }}^{{\text{T}}}Ey+{\mathrm{\pounds }}^{{\text{T}}}J+{\mathrm{\pounds }}^{{\text{T}}}G<0, if S\left({\text{y}}\right)<0 \end{array}{\text{G}}={\text{Eh}}+{\text{J}}.\right.$$

From Eqs. (17) to (22), the modified structure concept is applied to the slider controller for identifying the sunlight structure slider regions. The slider region S(f) values should be in the bounded condition. So, the overall structure works in variable sunlight insolation conditions as mentioned in Eq. (23). The sliding regions require state trajectories which are evaluated from the surface conditions. For evaluating the converter states which are closely near to the slider surface then the controller operation is given in Eq. (25). From Eqs. (22) to (25), the overall controller working conditions are mentioned in Eq. (26). The design variables of the controller are mentioned in Table 3, and the working of the adaptive modified slider controller is mentioned in Fig. 6. From Fig. 6, the converter network produces more distortions which are utilized in this network for running the sunlight MPP position at the actual MPP place. In this controller, the integrator, plus the filter network are combined to suppress the fluctuations of sunlight power. The high noise frequency values are eliminated by applying the gradient (Δ) value. From the controller structure, the error signal is associated with the sine value for tracing the global MPP place. The parameter ${{\text{V}}}_{\mathrm{Peak }}^{{\text{re}}}$ is forwarded to the slider network for evaluating the converter duty values range. The selected signals to the sliding system are sunlight insolation, sunlight supply voltage, plus sunlight current. Here, with the continuous fluctuations of solar insolation, the slider maintains the constant MPP position.

**Table 3:** Design values of adaptive modified slider controller structure.

S. no	Names of variables	Values
1.	Filter circuit generated output signal value (C(s))	1.0057
2.	The integrator of the slider controller utilized gain (K_c_)	6.2719
3.	Selected compensator value at leading factor (A(s))	0.382
4.	The surface constraint of the slider at horizontal condition (£_1_)	4.4431
5.	The surface constraint of the slider at vertical condition (£_2_)	0.285
6.	The entire system functioning frequency at diverse Sunlight systems (ω)	99.982 rad/sec
7.	The entire system higher cutoff functioning frequency (${\omega }_{h})$	9.9879 rad/sec
8.	Overall system sliding region value (Δ)	2.221
9.	Inverter sliding state of power semiconductor device (${\varnothing }_{1})$	0.182
10.	Inverter sliding state of power semiconductor device (${\varnothing }_{2})$	0.224
11.	Inverter sliding state of power semiconductor device (${\varnothing }_{3})$	0.2818
12.	Inverter sliding state of power semiconductor device (${\varnothing }_{4})$	0.4870

## Development of power inverter circuit for solar PV

**Figure 7:**
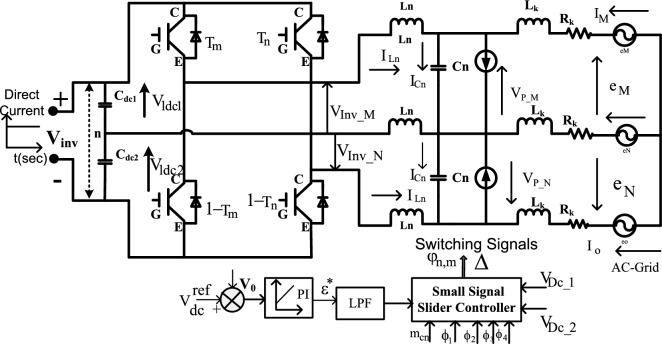
Overall inverter circuit for sunlight power fed 3-phase grid network.

**Figure 8:**
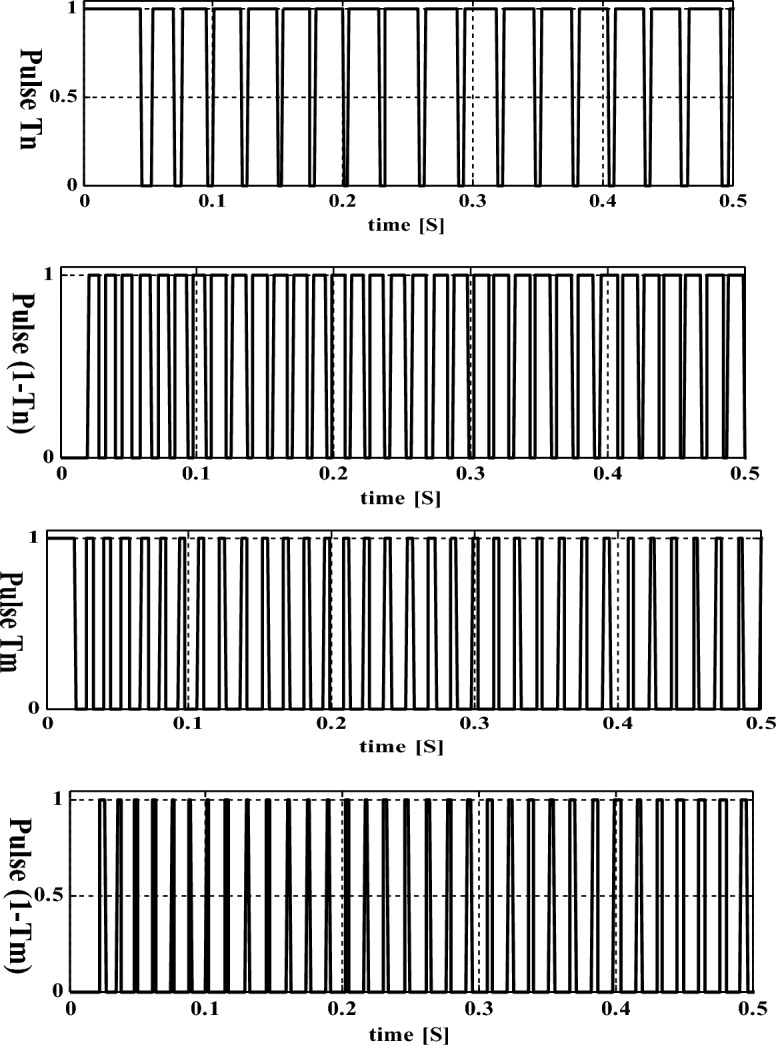
Switches operating states at quick changes of sunlight values.

The available supply of the converter circuit may not be fed directly to the grid network. So, the power transformation has been made by selecting the DC-AC conversion circuit. In the article^23^, the authors discussed the 3-leg bridge circuit for obtaining the three-phase power which is interlinked with the household, and gird power networks. However, this type of 3-leg circuit is not flexible for the uniform power supply to the central grid network because if any one of the switches fails, the entire power production network fails^24^. As a result, the overall network heating losses, plus harmonics losses are increased. In this proposed grid network, a 2-leg topology is introduced to eliminate the discontinuity in the grid supply power which is shown in Fig. 7. From Fig. 7, the first leg of the inverter circuit is interlinked with the phase (m), and the 2nd leg of the circuit is interfaced with the grid phase (o). Finally, the middle phase of the grid network is connected to the neural point of the DC-AC circuit which is named ‘n’. The design constraints of this circuit are mentioned in Tables 2 and 3, and its functioning pulses are illustrated in Fig. 8. From Fig. 8, the devices T_m_, plus 1–T_n_ start functioning at a time to supply the voltage V_Inv_M_. Also, the devices T_n_, plus 1–T_m_ work in the opposite way when associated with the previous state of operation. The voltages V_P-M_, plus V_P-N_ are appeared between the transmission lines ‘M’, and ‘N’, and its RMS value is ‘e’. The circuit L_n_-C_n_ helps eliminate the 3rd-order harmonics at non-uniform insolation values.

### T-4 switching pulses generation by using slider technology

From Fig. 7, the T-4 circuit starts functioning by interconnecting the slider block. The slider circuit receives all the inverter circuit state variables which are defined as m_cn_, ${\varnothing }_{1}, {\varnothing }_{2}, {\varnothing }_{3}, {\varnothing }_{4}$ plus V_DC-1,2_. Here, the DC-link circuit voltages are helpful for equal power distribution to the grid network. The supply side inverter circuit voltage error voltage is identified by comparing it with the reference voltage V^ref^. The inverter supply error voltage is applied to the slider block to suppress the fluctuations of dc-link voltages thereby the grid tries to work at the accepted power factor of the load. The LPF network helps the grid circuit maintain the uniform RMS voltages with a unity power factor. The term “ε” is adjusted continuously for supplying the balanced power to the local consumers. The mathematical representation of the inverter circuit, angle of displacement, plus overall grid angle of impedance is derived in Eq. (30). The functioning states of the DC-AC circuit are discussed in Table 4.

**Table 4:** Switching operation of two-leg three-phase inverter.

Contacts		States of IGBT functioning				Available voltages of inverter		
g	h	T_1_	T_2_	T_3_	T_4_	V_inv_M_	V_inv_N_	V_inv_O_
One	Zero	One	One	Zero	Zero	V_e_	− V_e_	0
One	One	One	Zero	One	Zero	V_inv_e_/3	V_inv_e_/3	− 2 V_inv_e_/3
Zero	Zero	Zero	One	Zero	One	− V_inv_e_/3	− V_inv_M_/3	2V_Inv-e_/3
Zero	One	Zero	Zero	One	One	− V_e_	V_e_	0

27 $$\frac{{{\text{dI}}}_{{\text{m}}}}{{\text{dt}}}=\frac{2}{3{{\text{L}}}_{{\text{n}}}}\left({{\text{e}}}_{{\text{p}}-{\text{m}}}-2{{\text{R}}}_{{\text{k}}}{{\text{I}}}_{{\text{m}}}-{{\text{V}}}_{{\text{pm}}}-{{\text{I}}}_{{\text{m}}}{{\text{R}}}_{{\text{k}}}\right)-{{\text{I}}}_{{\text{v}}},$$

28 $${{\text{I}}}_{{\text{v}}}=\frac{1}{3{{\text{L}}}_{{\text{n}}}}\left({{\text{e}}}_{{\text{n}}}-2{{\text{R}}}_{{\text{k}}}{{\text{I}}}_{{\text{m}}}-{{\text{V}}}_{{\text{p}}-{\text{m}}}-{{\text{I}}}_{{\text{p}}-{\text{m}}}{{\text{R}}}_{{\text{k}}}\right),$$

29 $$\frac{{{\text{dI}}}_{{\text{inv}}-{\text{dc}}}}{{\text{dt}}}=\frac{1}{{{\text{C}}}_{{\text{n}}}}\left({{\text{I}}}_{{\text{p}}-{\text{n}}}+{{\text{I}}}_{{\text{inv}}-{\text{n}}}-{{\text{I}}}_{{\text{p}}-{\text{m}}}\right),$$

30 $$\frac{{{\text{dV}}}_{{\text{cd}}1}}{{\text{dt}}}=\frac{-1}{{{\text{C}}}_{{\text{d}}1}}\left({{\text{u}}}_{{\text{a}}}{{\text{I}}}_{{\text{inv}}-{\text{a}}}+{{\text{u}}}_{{\text{b}}}{{\text{I}}}_{{\text{inv}}-{\text{b}}}\right)+\frac{{{\text{I}}}_{{\text{mag}}}\left(1-{\text{u}}\right)}{{{\text{C}}}_{{\text{d}}1}\left(1+{\text{N}}\right)},$$

31 $$\frac{{{\text{dV}}}_{{\text{dc}}-2}}{{\text{dt}}}=\frac{1}{{{\text{C}}}_{{\text{dc}}-2}}\left(\left(1-{\upvarepsilon }_{{\text{m}}}\right){{\text{I}}}_{{\text{inv}}-{\text{m}}}+\left(1-{\upvarepsilon }_{{\text{n}}}\right){{\text{I}}}_{{\text{inv}}-{\text{n}}}\right)+\frac{{{\text{I}}}_{{\text{m}}}\left(1-\upvarepsilon \right)}{{{\text{C}}}_{{\text{dc}}-2}\left(1+{\text{N}}\right)},$$

## Discussion of simulation results

The sunlight network is developed by considering the 3-diode solar system because its features are more efficient, highly accurate power, and current characteristics. Also, this circuit fill factor is little high when associated with the 1-diode, plus 2-diode topologies. The design constraints of the sunlight network are mentioned in Table 1. The parameter C_x_ helps maintain the uniform solar voltages irrespective of the sunlight insolation and its equivalent value is 10mF. The converter interlinked inductors support the solar system for enhancing the voltage profile of the PV array, and their design variables are defined in Table 2. Here, the utilized sunlight captured insolation values are 1000, 750, plus 500W/m^2^.

**Figure 9:**
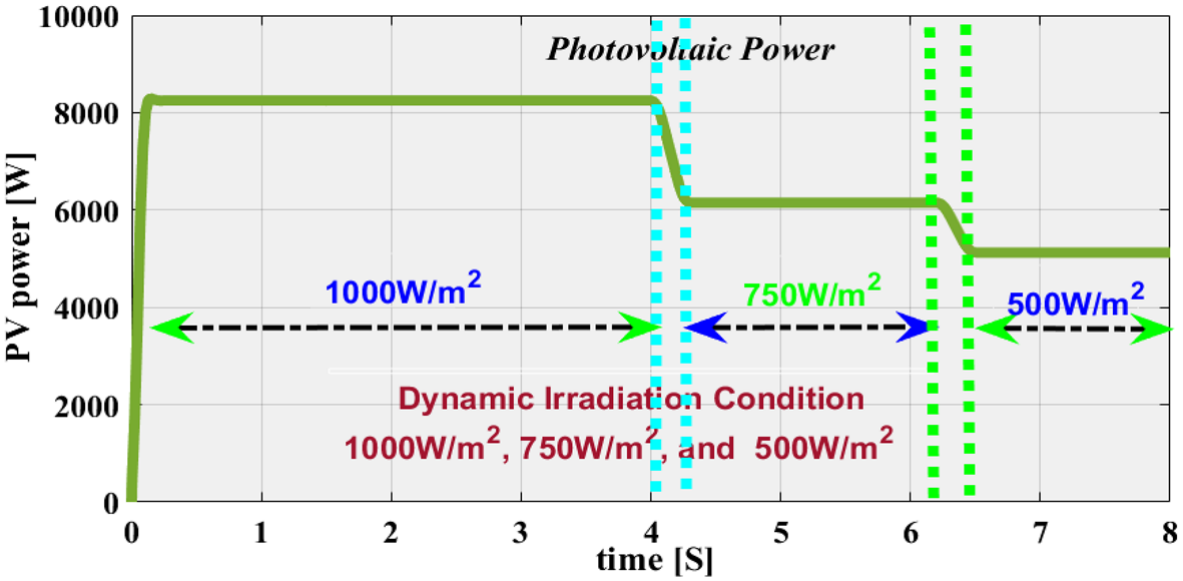
Extracted sunlight network power by utilizing the 3-diode solar PV circuit.

At uniform sunlight insolation, the extracted sunlight system power, solar voltage, plus PV currents are 96.82.99 kW, 0.4150 kV, and 0.02311kA respectively. The availed sunlight power at rapid variations environmental insolation is explained in Fig. 9. From Fig. 9, when the irradiations fall from uniform to 750W/m^2^ then the stabilizing time is 0.42 s. Also, the power gets reduced from 96.82.99 to 6.8289 kW. In addition, the voltage at this irradiation value is 405.89 V, plus the current supplied by the PV network is 16.18A. Finally, the availed sunlight network voltage at 0.5 kW/m^2^ is 401.98 V, and its related solar system current, plus PV is 11.897A, plus 4.782 kW.

**Figure 10:**
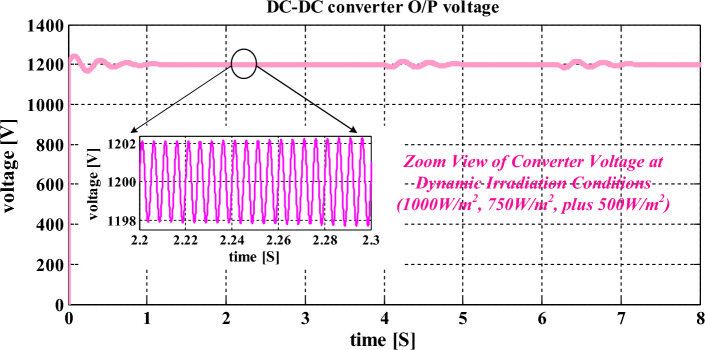
Converter network produced voltage signal at quick change of sunlight values.

From Fig. 10, the utilized reference voltage is 1200 V which is associated with the actual available voltage of the converter output signal. Here, the inverter circuit output linked capacitors collect the sunlight voltage with equal load distribution. Here, both the capacitors' C_ldc-1_, plus C_ldc-2_ voltages are equal to 599.891 V at quick changes of sunlight values which indicates that the neutral point of the proposed inverter circuit is constant, and balance the dc-link voltages of the capacitors. The balancing of neutral points helps the overall inverter network from the quick variation of PV network voltages. Also, the power of semiconductor devices damage possibility is reduced. So, the overall network functioning cost is optimized with the help of slider methodology. Here, the slider receives the sunlight voltages, and solar currents for producing the suitable duty value for the interlinked inductor converter circuit. The stabilizing time of the converter circuit voltage is 0.035 s which is an acceptable value for all sunlight temperature conditions. The evaluated modified slider power point tracking controller efficiency is 95.6% at variable sunlight values.

**Figure 11:**
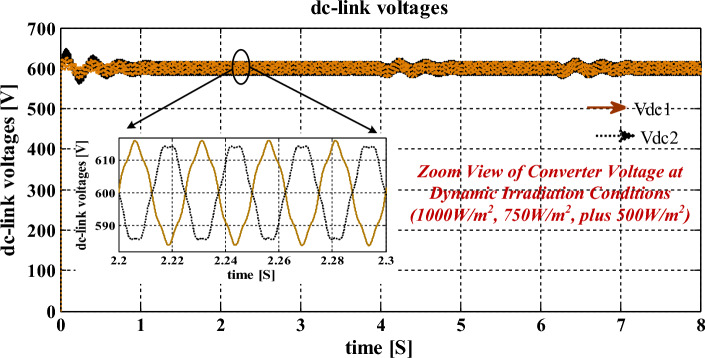
Balanced interlinked inverter output capacitors voltage signals at multiple insolation values.

**Figure 12:**
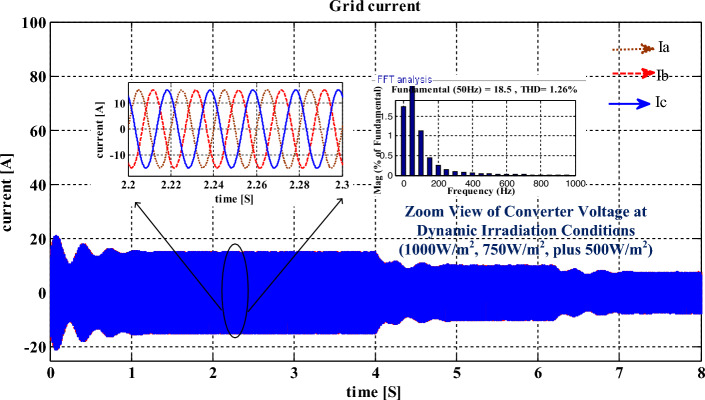
The available generated grid network 3-phase currents at diverse insolation values.

**Figure 13:**
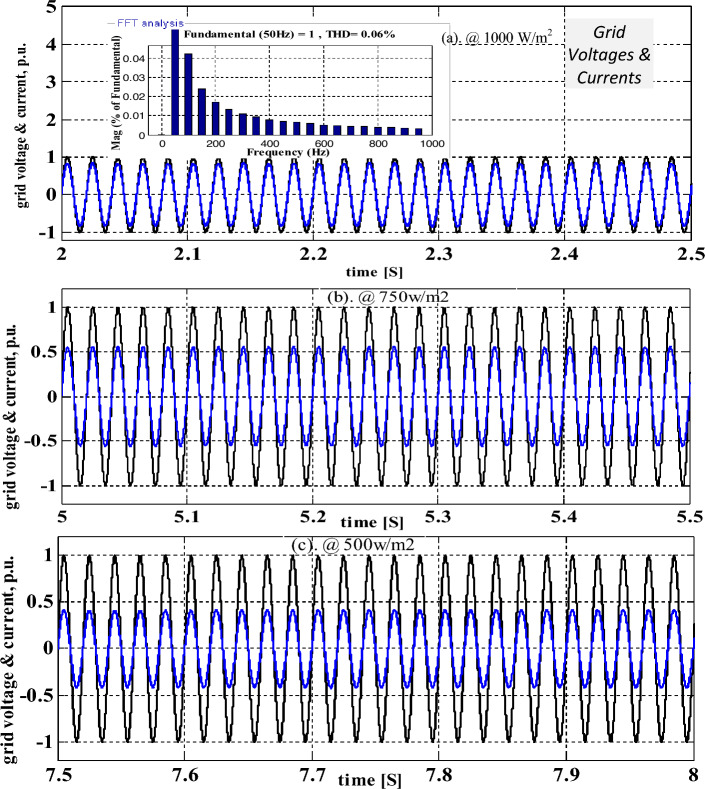
Available per unit grid network current, plus inverter supplied voltages.

The distortions content involved in the converter voltage is 2 V. From Fig. 11, the voltage of the interlinked capacitor varies between 604.89 and 598.22 V, and it’s almost the same. However, the inverter-fed grid network currents at 1 kW/m^2^ are 18A, and its available current at 0.75 kW/m^2^ is 14.78A. Finally, at 0.5 kW/m^2^, the grid network current is 9.759A respectively. At a functioning frequency of 50 Hz, the grid supplied total harmonic content is 1.26% which is very low and it is evaluated by the interlinking of the modified slider technique. The obtained 3-phase grid available currents under diverse atmospheric conditions are illustrated in Fig. 12. Finally, the per unit voltages are considered because of the easy analysis of the overall sunlight-fed grid network system as mentioned in Fig. 13. The maximum THD value of this grid currents and load voltages is 0.06%. So, the modified slider block is very useful for the B-4 converter circuit to produce electrical energy at the unity power factor.

## Conclusion

The triple diode sunlight circuit is considered for the development of PV modules because of its more accurate nonlinear curves, most effective in operating efficiency, plus more power extraction. In the first objective, a new interlinked inductor DC-DC power network is developed to reduce the installation price of the sunlight system. These converter features are wide source voltage, more voltage conversion ratio value, low passive components utilization, less catchment area needed, plus more flexibility. However, the duty signal production for the converter, plus the operation of inverter circuits at shaded conditions of the sunlight system is a little challenging task. So, the modified slider methodology is introduced in the 2^nd^ objective for extracting more sunlight power and supplying the energy to the local networks and grids at unity power factor. The features of this proposed MPPT controller are fast MPP identification, low design complexity, easy operation, plus good understanding. Finally, in the third objective, the B-4 inverter circuit is proposed for uniform energy supply to the loads at all types of atmospheric conditions.

## Data Availability

The data used to support the findings of this study are included in the article.
